# Using Avoidant Emotion-Focused Coping and being a Woman with Adverse Childhood Experiences as the Worst-Case Scenario for Internalising Problems

**DOI:** 10.1007/s40653-025-00688-z

**Published:** 2025-02-08

**Authors:** Aitana Gomis-Pomares, Laura Lacomba-Trejo, Lidón Villanueva

**Affiliations:** 1 Developmental and Educational Psychology Department, Universitat Jaume I, Castellón, Spain; 2 Developmental and Educational Psychology Department, Universitat de València, Valencia, Spain

**Keywords:** Adverse childhood experiences, Coping strategies, Internalising problems, Young adulthood

## Abstract

Adverse childhood experiences (ACEs) bring an increased risk for the development of internalising outcomes. Nevertheless, how the cumulative effect of ACEs combines with other variables, such as coping strategies, to give rise to internalising problems has been little studied so far. Therefore, the current study investigates which specific combinations of total ACEs, coping strategies, and sociodemographic variables influence depression, anxiety, and stress. To this end, 420 young Spanish adults (63% women) 18–20 years old (mean age = 18.92; *SD* = 0.77) participated in the study. Participants answered sociodemographic questions and completed the ACEs Questionnaire, the Depression, Anxiety, and Stress Scale, and the Brief Coping Scale. Both fuzzy-set qualitative comparative analysis and regression models suggested that the cumulative impact of ACEs, in combination with avoidant emotion-focused coping, may conduce internalising problems in women. These findings could serve as a basis for interventions aimed at the primary screening of populations more sensitive to the development of internalising problems as well as in the re-education of adaptive coping strategies in those who have suffered ACEs, in order to avoid more severe long-term problems.

## Introduction

Adverse childhood experiences (ACEs) have appeared as an important developmental factor that negatively influences life course trajectories (Anda et al., 2006; Felitti et al., 1998; Muñoz et al., 2024; Scully et al., 2020). It was estimated that nearly 67% of the population have experienced at least one ACE, while 10% have experienced five or more (Bellis et al., 2014; Felitti et al., 1998). These exposures are shown to be related to a wide range of negative outcomes like antisocial behaviour (Basto-Pereira et al., 2022; Gomis-Pomares & Villanueva, 2020) or drug use (Dube et al., 2003) among adults Leza et al., 2021; Schwartz et al., 2024) in a dose–response fashion. Nonetheless, studies have also demonstrated a particularly strong relationship between ACEs and internalising outcomes (Lindert et al., 2014; Lippard & Nemeroff, 2020).

According to the World Health Organization (WHO, 2022), about 970 million people have a mental disorder. In Spain, anxiety disorder is the most frequent mental health problem, affecting 6.7% of the population and remaining stable across age groups. Depressive disorder is the second most frequent, being present in 4.1% of the population and growing progressively up to the age of 75 (Clinical Records from Primary Care [BDCAP], 2021). In the case of children and adolescents, the consequences of these disorders are worse, as they can interfere with normal brain development and social functioning (Milojevich et al., 2018).

Previous research in this field has investigated the association between ACEs and an increased risk for anxiety disorders (Gibb et al., 2007; Sareen et al., 2013), depressive disorders (Tan & Mao, 2023), and stress disorders (Crede et al., 2023) in adulthood. However, only recent research has begun to focus on the study of ACEs and internalising behaviours in children and young people (Elmore & Crouch, 2020), even when evidence shows that a first mental disorder occurs before the age of 14 in 33% of cases, and before the age of 18 in almost 50% of cases (Solmi et al., 2021).

One of the main contributing factors to the risk of internalising problems is the experience of multiple adversities in childhood and adolescence (Gomis-Pomares & Villanueva, 2022). In this regard, during childhood an ACE score of 4 or more was associated with a 7.3 times greater likelihood of developing anxiety and depression, compared to individuals with less than four ACEs (Hunt et al., 2017; Lew & Xian, 2019). Moreover, children between the ages of 6 and 17 who had experienced many ACEs were 3 times more likely to suffer from anxiety or depression and 4 times more likely to report a comorbid history of both internalising behaviours (Lew & Xian, 2019).

Additionally, premature exposure to ACEs has proven to increase people’s risk for exhibiting deficits in emotional functioning, influencing how individuals cope with risky situations and regulate their emotions (Cicchetti & Rogosch, 2009). Therefore, coping strategies can emerge as the cognitive system moderating the relationship between ACEs (stressors) and stress responses (Gartland et al., 2012; Pearlin, 1991). In other words, ACEs may impact how individuals cope with stressful events, which in turn, may prolong, worsen, or, conversely, ameliorate the stress response (Wadsworth, 2015).

According to the stress response model (Connor-Smith et al., 2000), children's first attempts at self-regulation begin in infancy, in reciprocal coregulation with the primary carer. Hence, the presence of extremely stressful events or inappropriate caregivers disrupts the sequence of normative development and causes young people and adults to continue to use maladaptive coping strategies, such as avoidance or denial (Zimmer-Gembeck et al., 2011). Similarly, cognitive theories of depression (e.g., Beck, 1967) suggest that children develop negative coping and thinking patterns from early invalidating interactions with caregivers. Thus, the repeated use of maladaptive forms of coping, as well as a lack of exposure to healthy alternatives, or repeated exposure to a strong stressor, can lead to the solidification of an inappropriate coping style (Segrin et al., 2013).

Despite the existence of multiple definitions, coping strategies are most commonly defined as cognitive and behavioural efforts made in response to a threat (Lazarus & Folkman, 1984), or, in other words, as intentional responses to a stressful life event (Compas et al., 2017). Sheffler et al. (2019) distinguished between two strategies which are frequently related to ACEs: problem-focused (PF) coping (i.e., a positive problem-solving strategy based on managing stress by directly addressing the source of the problem or stressor), and avoidant emotion-focused (AEF) coping (i.e., a negative coping strategy based on managing stress or difficult emotions by trying to avoid or suppress feelings), (Lazarus & Folkman, 1984). In this sense, studies have indicated that individuals who have experienced ACEs will present lower PF coping (Gipple et al., 2006) and higher AEF coping (Leitenberg et al., 2004). This is especially noticeable in adolescence and emerging adulthood, a period where individuals deal with different life changes that may require an increasingly sophisticated set of coping and regulatory skills (Aldao & Nolen-Hoeksema, 2013), which, in turn, may be lacking in populations that have experienced childhood maltreatment.

Apart from that, coping strategies as well as associated internalising disorders seem to differ between some sociodemographic variables, such as sex (Gomis-Pomares & Villanueva, 2022). The life-long incidence of depression in women is about 21% compared with 13% in men (Zender & Olshansky, 2009). Anxiety disorders are diagnosed twice as often in women (Campanella et al., 2012). Besides, men and women are often thought of as having different coping styles. For instance, men are more likely to confront a problem head-on (Pearlin & Schooler, 1978), while women show a more emotional response to problems and spend more time talking about them with friends or family (Folkman & Lazarus, 1980). However, sex differences in coping have not yet been conclusively established (Tamres et al., 2002).

Another sociodemographic factor considered in previous literature is socioeconomic status (SES). People of lower SES may be particularly at risk of using ineffective solutions as they have little money to solve problems, may sometimes have little to lose by resorting to a negative or even criminal coping strategy, or may be more likely to have personal traits (such as low self-control) that make coping ineffective (Agnew, 2006). Moreover, the relationship between economic hardship and mental health problems is unclear: Some studies have found the greatest risk of poor mental health in participants experiencing economic hardship (Everson et al., 2002), while others found an increase in depression rates in both the lowest and highest income groups (Weinberger et al., 2018).

Hence, the present study seeks to address these gaps by exploring how different coping mechanisms may mitigate or exacerbate the long-term negative effects of ACEs, highlighting potential sex differences in coping strategies, and investigating the complex interactions between sociodemographic factors, ACEs, and coping strategies. Additionally, the study focuses on emerging adulthood, a critical developmental period distinct from adolescence and later adulthood, as it represents a time of heightened vulnerability and opportunity for intervention. By conducting the research within a Spanish population, the study contributes to the understanding of these dynamics in a cultural context that has been underrepresented in ACEs research. To reach this, different methodologies of analysis were used: regression models and fuzzy-set Qualitative Comparative Analysis (fsQCA). The latter methodology makes it possible to emphasise the most relevant combinations of variables that lead to the same outcome (Ragin, 2014), thus enriching the understanding and comprehension of the results obtained.

To achieve this purpose, the primary goal of the present study was to explore the influence of sociodemographic variables (sex and SES), ACEs (cumulative effect), and two different kinds of coping strategies (PF and AEF coping) on the development of internalising outcomes (anxiety, depression, and stress). Additionally, the specific combinations of these variables that lead to a specific internalising result were assessed by means of an fsQCA. The posited hypotheses were (1) the cumulative effect of ACEs will increase the odds of internalising problems; (2) the use of maladaptive coping strategies (i.e., AEF coping) will boost internalising problems; and (3) female sex as well as low SES will predict internalising outcomes.

## Method

### Participants

Participants in this study were 420 young adults aged between 18 and 20 years (63.3% women), with a mean age of 18.92 (*SD* = 0.77), from the Valencian Community in Spain. The sample was predominantly Spanish (92.7%), with a small representation from ethnic minorities (7.3%). Specifically, 2.3% of participants were of Romanian origin, 1.9% were Latin American, 1% were of African origin, and 2.1% were from other backgrounds. Of the total sample, 4.3% of the participants completed primary education studies, 42.7% completed secondary school studies, and 53% had completed or were university students. Per SES, 29.2% had a low SES level, 54.7% had a medium SES level, and 16.1% had a high SES level.

SES was coded based on different criteria depending on whether individuals were financially dependent on their parents or financially independent. For individuals who were financially dependent, SES was determined by the education level and profession of their parents. For those who were financially independent, SES was assessed based on their own education level and profession. SES was categorised as high, medium, or low based on these factors: individuals in managerial positions within large companies or professions requiring advanced education were classified as high SES; those in small businesses, specialised professions, or with a high school education were classified as medium SES; and individuals holding non-specialised jobs or those that only require basic studies were categorised as low SES. In this study, the interrater reliability was deemed strong, with an average value of 0.85.

### Instruments

The Adverse Childhood Experiences Questionnaire (ACEs Questionnaire) developed by Felitti et al. (1998) was used to assess 10 categories, namely sexual abuse (“*Were you touched of fondled in a sexual way?*”); physical abuse (“*Were you hit so hard that you had marks or were injured?*”); emotional abuse (“*Do you believe you were emotionally mistreated?*”); physical neglect (“*Was there someone who took you to the doctor when you needed it?*”); emotional neglect (“*Were you sworn at, insulted, or belittled?*”); living in a household with domestic violence (“*Was someone pushed, grabbed, slapped, or had something thrown at them?*”); parental divorce (“*Did your parents separate or divorce at any time?*”); household substance abuse (“*Have you lived with someone who used street drugs?*”); mental illness in the household (“*Was someone in your home depressed or mentally ill?*”); and incarceration of a household member (“*Did someone in your home go to prison?*”). The areas of emotional and physical abuse, neglect, and witnessing domestic violence were evaluated on a Likert scale ranging from 0 to 4. Meanwhile, the rest of the ACEs were assessed dichotomously (Yes/No). Each ACE dimension was dichotomised according to the original author’s instructions (Felitti et al., 1998; Pinto et al., 2014). A total ACEs score was calculated by adding all the present ACEs. The questionnaire showed good psychometric properties (Murphy et al., 2014; Pinto et al., 2014).

The Depression, Anxiety, and Stress Scale (DASS-21) (Daza et al., 2002) was used to assess symptoms of three emotional states in young adults: anxiety, depression, and stress. These emotional states consist of three Likert-type subscales with four response options (0–3) and seven items each, resulting in a total of 21 items. Item examples include: “*I felt that my hands were trembling*”, “*I noticed that I was agitated*”, or “*Sometimes I get annoyed when things do not go my way*”. To obtain the total score for each subscale, the mean of the items of each subscale was calculated. The questionnaire demonstrated adequate psychometric properties (Daza et al., 2002; Ribeiro et al., 2004).

The Brief Coping Scale (BCS) was used to assess coping responses in adults (Carver, 1997; Perczek et al., 2000). The scale includes responses that are presumed to be adaptive and functional (e.g., emotional support or active coping) and responses suspected of being maladaptive (e.g., self-blame or substance abuse). It comprises 14 dimensions or coping strategies, each of which consists of two items. In general, each dimension should be analysed separately to evaluate its relationship with the other variables, as there is no global score. The following classification of coping strategies proposed by Sheffler et al. (2019) were used in this study: PF coping and AEF coping. PF coping was the sum of three subscales, “positive reinterpretation and growth”, “active coping”, and “planning”. For example, one of the items used in the PF dimension was: “*I take additional action to try to resolve the problem*”. AEF coping comprised the following three subscales: “focus on venting of emotion”, “denial”, and “behavioural disengagement”. One example item of the AEF dimension was: “*I feel great emotional distress and find myself expressing those feeling frequently*”. Responses are measured on a 4-point Likert scale (1–4), where a higher score indicates a greater use of coping strategies. The questionnaire previously demonstrated good psychometric properties (Perczek et al., 2000).

### Procedure

The data were collected as part of an international study, the SOCIALDEVIANCE1820 Research Project, focusing on pro/antisocial behaviour in young adults (Basto-Pereira et al., 2020). After obtaining consent from the university's ethics committee (reference number 22/2018), participants were recruited using convenience and simple snowball sampling methods from different locations, including workplaces, sports organizations, universities, high schools, and adult schools. The researchers explained the objectives of the study, emphasising that all questionnaires presented were completely anonymous and confidential, and that participation was voluntary. Subsequently, with the participants' consent, the different self-report questionnaires were administered. The participants had the chance to enter a prize draw by completing the questionnaires.

### Data Analysis

Descriptive statistical analyses were conducted for the variables under study, and linear regression models were performed using SPSS software. Hierarchical regression models were conducted to analyse the predictive ability of the variables under study, with the three dimensions of the DASS (anxiety, depression, and stress), the total DASS score, as the dependent variables, and total ACEs, sex, SES, PF coping, and AEF coping as the predictor variables. The model consisted of three different steps (Table 1). In the first step, sex and SES were included. In the second step, the total number of ACEs was included, and in the third step, the PF coping and AEF coping variables were included.

**Table 1:** Hierarchical regression model of internalising problems

Predictor	DASS anxiety				DASS depression				DASS stress				DASS total			
	*ΔR*^*2*^	*ΔF*	β	*t*	*ΔR*^*2*^	*ΔF*	β	*t*	*ΔR*^*2*^	*ΔF*	β	*t*	*ΔR*^*2*^	*ΔF*	β	*t*
Step 1	.03	6.39			.04	8.48			.05	10.42			.05	10.10		
Women			.12	1.81			.08	1.27			.24	3.39			.15*	2.46
SES			−.15*	−3.18			−.19*	−3.98			−.16*	−3.23			−.17*	−3.89
Step 2	.02	8.67			.07	29.86			.02	10.69			.04	19.03		
ACEs Total			.06*	2.94			.10*	5.47			.07*	3.27			.07*	4.36
Step 3	.12	29.29			.11	29.03			.11	27.31			.14	36.79		
PF			.01	.69			-.03*	−1.92			-.01	-.95			-.01	-.83
AEF			.12*	7.40			.12*	7.59			.13*	7.39			.12*	8.55
*Durbin-Watson*	.76				.93				.74				.45			
*R*^*2*^_*adj*_	.16				.21				.17				.22			

Note: *ΔR*^*2*^ = Change on* R*^*2*^*; ΔF* = Change on *F;* ß = regression coefficient; *t* = t value; ^*^p. ≤ *.05*

*Note. * Socioeconomic status (SES); Adverse Childhood Experiences (ACEs); Problem-focused coping (PF); Avoidant emotion-focused coping (AEF)

FsQCA was also performed to predict the main combinations of the presence and absence of anxiety, depression, stress, and the total DASS score. Missing data were removed, and variables were recalibrated between 0 and 1 when necessary, taking into account whether the variable had only two values: 0 represented the absence of the characteristic, and 1 its presence. With continuous variables, an automatic recalibration was performed with the fsQCA software that considered three thresholds: 0% (low level or totally out of the set), 50% (intermediate level, neither in nor out of the set), and 90% (high level or totally in the set), (Woodside, 2013). Also, based on previous literature (Gomis-Pomares & Villanueva, 2020), in the case of ACEs, the variable was manually recalibrated, considering 0 adverse experiences at 10% (low level or totally out of the set), between 1 and 3 at 50% (intermediate level, neither in nor out of the set), and more than 4 (experiences) at 90% (high level or totally in the set).

On the one hand, an analysis of the necessary causes for the presence or absence of each of the predicted variables was carried out, considering that a condition is necessary when its consistency is ≥ 0.90 (Ragin, 2008). This was followed by an analysis of sufficient conditions for the high and low levels of each of the dependent variables. “Sufficient conditions” meant that, although the conditions are not always present for a result to occur, they can lead to this result. In this sense, a model is informative when the overall consistency is around or above 0.74 (Eng & Woodside, 2012).

A sufficiency analysis offers three possible solutions (complex, parsimonious, and intermediate). The combination between the intermediate and the parsimonious solution is presented. For this purpose, the intermediate solution is shown, and those conditions that appear in the parsimonious solution are identified. In this way, the core conditions are obtained (  ,  ). The remaining conditions presented are the peripheral conditions (  ,  ). As can be seen, core conditions are represented as a larger size, as indicated (Pappas & Woodside, 2021). These QCA models also allowed us to identify the percentage of variance explained, or the cases in which the model was satisfied (i.e., the coverage and goodness-of-fit indicators [consistency]) (Eng & Woodside, 2012; Ragin, 2008).

## Results

### Hierarchical Regression Models

Hierarchical regression models with the internalising outcomes are shown in Table 1. In the first step, only SES showed a significant predictive relationship with all the internalising problems (stress, anxiety, and depression), as well as with the total DASS score, showing that a low SES was related with a higher probability of presenting internalising problems. Additionally, sex also had a significant predictive relationship with the total DASS score, with women presenting a higher risk of suffering from them (an explained variance ranging 3–5%). In the second step, the ACEs total score showed a significant predictive relationship with all internalising problems, as well as with the total score of ACEs, the explained variance ranging 2–6%. Finally, in the third step, PF coping showed a significant negative predictive relationship only with depression, while AEF coping showed a significant predictive relationship with all internalising problems and their total score (explained variance percentage ranging 10–13%). It was evident that there was a substantial increase in the total variance explained, compared to steps 1 and 2. The final explained variability for the models was 16% for anxiety, 21% for depression, 17% for stress, and 22% for the total DASS score.

### Fuzzy-set Qualitative Comparative Analysis (fsQCA)

#### Analysis of Necessity

Descriptive and calibration analyses of the fsQCA were performed. There was no condition necessary to predict high and low levels of anxious, depressive, and stress symptoms (consistency < 0.90) (Ragin, 2008).

#### Analysis of Sufficiency

FsQCA models are informative when their consistency is around 0.74 (Eng & Woodside, 2012). Regarding high levels of anxiety, three paths explained 31% of the cases. The most relevant combination considered low levels of SES, the presence of ACEs, and the presence of AEF coping styles. The next pathway resulted from the combination of being female, having ACEs, the presence of AEF coping styles, and the absence of PF coping styles. The third and final pathway considered being male, with low SES, and with AEF and PF coping styles.

On the other hand, a single pathway was found to explain 23% of the cases of low anxiety levels. This pathway resulted from the combination of the presence of high SES and PF coping strategies, with the absence of ACEs and AEF coping strategies.

Conversely, an explanatory pathway for high levels of depression was found that predicted 23% of cases and considered being female and the presence of ACEs, AEF coping, and low PF coping. In explaining low levels of depression, two possible pathways that predicted 47% of the variance were found. The first pathway resulted from the combination of high SES, the absence of ACEs, and the absence of AEF coping. The second pathway combined being female with exhibiting PF coping, the absence of ACEs, and the absence of AEF coping.

In predicting high levels of stress, two pathways were found that explained 39% of the cases. The first or most relevant pathway resulted from the combination of low SES, being female, and presenting AEF coping. The second pathway combined being female with ACEs, and with AEF coping. Regarding low levels of stress, only one pathway was found that explained 17% of the cases of low levels of the variable, including being male, having low levels of AEF and PF coping, and reporting high SES.

In explaining the high levels of total DASS scores, two possible pathways were obtained that explained 28% of the variance. The most relevant one combined being female, having low SES, the presence of ACEs, and using an AEF coping strategy. The second pathway resulted from the interaction between being female, high ACEs, a low presence of PF coping, and a high presence of AEF coping. Per explaining low levels of distress, three pathways were obtained that explained 52% of the cases. The most relevant pathway accounted for the combination of high SES with the absence of ACEs, while using AEF coping strategies. The second pathway resulted from being male and reporting no ACEs and no AEF coping. The last pathway included being male without using PF or AEF coping strategies (Table 2).

**Table 2:**
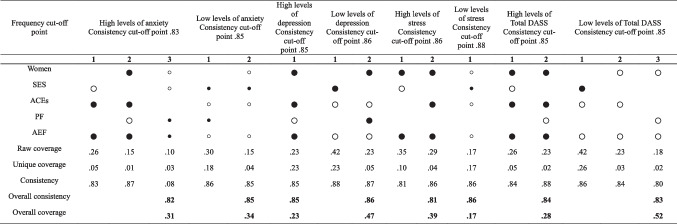
Sufficiency analysis for high and low levels of internalising problems

Note:. Socioeconomic status (SES); Adverse Childhood Experiences (ACEs); Problem-focused coping (PF), Avoidant emotion-focused coping (AEF); Expected vector according to Fiss nomenclature (2021); For high levels of anxiety; depression and stress: 1,0,1,0,1; For low levels of anxiety, depression and stress: 0,1,0,1,0; For high levels of deviant behavior: 0,0,1,0,1; For low levels of deviant behavior: 1,1,0,1,0

## Discussion

In this study we explored the association between ACEs and later trajectories of internalising outcomes in 18- to 20-year-olds, while accounting for sociodemographic and coping strategies. We employed regression and fsQCA methodologies to analyse the variables that may influence these relationships.

First, in relation with the first hypothesis, we anticipated that the sum of ACEs would boost the odds of internalising outcomes. Both methodologies found evidence for this phenomenon. This relationship is of particular interest because it revealed a significant positive association between an increased number of ACEs and increased levels of anxiety, depression, and stress. Our findings are reminiscent of previous studies where a history of childhood trauma is evident in people with depression, anxiety, and stress (Crede et al., 2023; Gomis-Pomares & Villanueva, 2022; Sareen et al., 2013; Tan & Mao, 2023). According to cognitive theories (e.g., Beck, 1967), children develop negative strategies to protect themselves from overwhelming exposures to stressful situations (Ullman & Peter-Hagene, 2014). Thus, children who experiences maltreatment, who is continually invalidated, and who are exposed to negative messages (such as how “stupid” or “worthless” they are), will develop a self-schema full of such statements. Because of these types of messages or because of a lack of affection received, it would be more probable that these children develop negative thinking patterns which, in the long term, will give way to internalising problems earlier than individuals who are not exposed to this type of negative information directed at themselves. Besides, previous results showed that young adults are particularly sensitive to the presence of internalising problems (Kessler & Wang, 2008; Solmi et al., 2022). This heightened sensitivity may be closely tied to the significant transitions that typically characterise this life stage, as individuals move from adolescence into adulthood (Arnett, 2000). During this period, young adults face critical tasks such as exploring their identity, seeking greater independence, and adjusting to evolving social roles and responsibilities. These developmental challenges often coincide with mounting academic or career pressures and the need to establish a sense of self, all of which can create a fertile ground for internalising issues to emerge (Blanco et al., 2008). For instance, a young adult navigating the pressures of academic performance, social belonging, and future career choices may experience heightened stress or self-doubt, increasing their vulnerability to internalising symptoms (Conley et al., 2014).

Consistent with our second hypothesis, the use of maladaptive coping strategies (i.e., AEF coping) increased the odds of developing internalising problems. A potential rational for this relationship may lie in the fact that ACEs profoundly impact how the brain processes stress, controls impulses, and responds to rewards (Nusslock & Miller, 2016). Previous studies had indicated that ACEs can induce structural changes in the brain due to chronic hyperactivation of the stress-response neural systems, with substantial ramifications for the functioning of the adult brain (Glaser, 2000; Horner & Hamner, 2002). In turn, these changes in the brain and mind can shape how stressful situations are perceived and how individuals cope with those tensions. Therefore, those exposed to ACEs not only tend to perceive situations as more stressful than others but may also struggle more to effectively manage those stresses. Collectively, these complex processes lead to the experience of more stressful situations, reacting more strongly to them, and having more difficulties dealing with those difficult moments (Nusslock & Miller, 2016; Sheffler et al., 2019).

Apart from that, and as previous studies indicated, it seemed that an additional relationship might arise among a cumulative measure of ACEs, the adoption of maladaptive coping strategies (Leitenberg et al., 2004), and a reduction in the adoption of PF strategies (Gipple et al., 2006). In this respect, maltreated children may tend to perceive their environment as threatening and unpredictable, thus generating a hostility bias and a perception of being unable to change the environment. Furthermore, ACEs may affect normal development and disrupt the development of emotion regulatory mechanisms (Ehring & Quack, 2010; Messman-Moore et al., 2010) such as the nonacceptance of one’s emotions, which in turn may mediate the effects of ACEs on internalising outcomes (Kim & Cicchetti, 2010). Stress models (Connor-Smith et al., 2000) might also support these results since an impaired normative development and poor emotional regulation – as a result of exposure to stressful situations and negative parental modelling – may be the result of the adoption of unripe coping strategies. In turn, the use of an avoidance coping strategy serves to maintain internalising disorders, as the person never confronts the stressor or situation. Likewise, non-exposure prevents the person from coping with the stressor and from "unlearning" the erroneous beliefs they have come to associate with the stressor.

Notwithstanding the foregoing, the current study results imply the importance of also considering the specific combination of variables that may lead to internalising responses. In this sense, both our analytic strategies showed the presence of ACEs, the adoption of AEF coping strategies, and being a woman as the main combination leading to internalising outcomes. That is, a woman who has suffered adversities in childhood and adolescence, and now, as a young adult is trying to cope with problems using denial, venting, or behavioural disengagement is at high risk of developing internalising problems. Basically, these individuals are not confronting or solving problems but trying to diminish their negative affective responses triggered by the stressor (Lazarus & Folkman, 1984). Moreover, some of these strategies have been associated with less perceived control or the feeling that the problem cannot be solved, therefore, leading to depression (Brown et al., 2007; Martin et al., 2021).

Some of these maladaptive strategies related to the emotional domain may be early on associated with the absence of affect regulation skills due to ACEs, that is, the lack of a proper context to develop them (Briere, 2002). Therefore, using this kind of strategy may be functional in the short term, in childhood, but continuing to use these less mature or primitive forms of coping, such denial, later in development limit the individual’s coping repertoire and contribute to the development of psychological problems (Wadsworth, 2015). These results support the strong connection between AEF coping strategies and ACEs, versus the not-so-significant role of PF coping in this relationship, as found in previous studies (Leitenberg et al., 2004; Sheffler et al., 2019). In fact, the presence of negative coping strategies seems to be more harmful than the absence of adaptive coping strategies (Aldao et al., 2010).

Third, per our last hypothesis which described that being female and having a low SES would increase internalising problems, differences were found. Regarding coping styles, women were more prone to use AEF coping while men were more prone to use PF coping strategies. The reasons for gender-related differences in coping strategies could depend on gender-roles that men and women internalise in society as well as the number of stressors that they generally deal with (Anderson & Leslie, 1991; Tamres et al., 2002). According to gender socialisation theory (Fagot et al., 2000), and because of the male stereotype, men should be action-oriented, direct, and assertive, and are therefore more likely to deal with problems by focusing on them. On the contrary, for women, it is socially acceptable to turn to others for help in times of stress, while men are discouraged, as that is a sign of “weakness” (Tamres et al., 2002). Apart from that, regarding low SES and its relationship to the development of internalising outcomes, individuals from lower socioeconomic backgrounds are less likely to access mental health services due to financial barriers, lower insurance coverage, and limited availability of resources in their communities (Verbeek et al., 2019). As suggested by previous authors (Caplan & Schooler, 2007; Gallo et al., 2005), individuals from lower SES groups often face a combination of structural and environmental challenges that can influence their access to coping resources. Limited access to mental health services, recreational opportunities, balanced diets, and other supportive resources may create additional stressors and restrict the range of coping strategies available. These conditions can make it more difficult to manage stress effectively and may increase reliance on coping mechanisms that are less adaptive. Furthermore, studies indicate that individuals from lower SES backgrounds are more likely to encounter barriers to accessing mental health care and preventive resources, which could contribute to a higher prevalence of mental health issues (Muntaner et al., 2004). Similarly, perceived control over life circumstances is often influenced by a range of systemic and social factors, which might partially explain the increased vulnerability to internalising problems observed in some individuals from lower SES groups.

Although the present study represents a significant step in investigating potential coping mechanisms that may contribute to long-term consequences of child maltreatment, it represents only one stage. Study limitations must also be acknowledged. First, this study focused on behavioural coping strategies. However, other psychological forms of coping (such as positive reinterpretation, planning, or the use of humour) should be considered to see if the same results are obtained (Litman, 2006). Second, the age range of our participants was limited (young adults between 18 and 20 years old). For this reason, the results cannot be generalised to other age groups. Nonetheless, the proximity of this age to childhood and adolescence allowed for a more accurate recall compared to older-age groups, which is an advantage of how our evaluation was carried out in the present work (i.e., self-reporting), (Pinto et al., 2014). Third, our analyses were based on cross-sectional data. This can be problematic when examining relationships that occur over time (Thoits, 1994). Therefore, longitudinal research would be required to gather more detailed information on the temporal relationships of the variables studied. Fourth, only the cumulative effect of ACEs was taken into account, however a differential approach would allow for a further investigation of the relationship of specific ACEs with coping strategies and internalising problems. In this sense, it is very likely that not all ACEs exert the same influence on this relationship. Furthermore, future studies should consider combining self-report questionnaires with other, more objective reporting measures (such as child protective services reports) in order to provide more accurate data on the impact of adverse experiences. Additionally, attention should be paid to creating positive childhood experiences (PACEs) that reflect and build resilience in children, families, and communities. Finally, the sample of young Spanish adults offers valuable insights that contribute to the growing body of research in this field. However, caution is necessary when attempting to generalise these findings to other populations given the reliance on convenience and snowball sampling methods.

## Conclusion

Despite its limitations, this study makes significant contributions to the literature. It addresses key gaps, including the association of ACEs, the use of coping strategies, and the influence of sociodemographic factors (such as sex and SES), in the prediction of internalising outcomes. By identifying these combinations, fsQCA helps clarify the specific relationships between these factors, offering a better understanding of how they interact to cause internalising problems. Additionally, the study was carried out with a Spanish sample of emerging adults, a developmental stage that is particularly significant for forecasting internalising outcomes, and within a cultural context that has been relatively underexplored. Moreover, the application of two distinct types of predictive analyses proved invaluable in enhancing our understanding of the variables associated with internalising problems.

Therefore, this study provides a clearer picture of the risk factors associated with internalising psychopathology and ideally allows for early detection and intervention. Being aware that women with high family stress, AEF coping strategies, and low SES are at particularly high risk for developing anxiety, stress, or depression allows healthcare providers, educators, or social workers to screen these individuals more effectively. On the other hand, establishing the consequences that coping strategies bring emphasises the imperative need to teach people more effective coping approaches (Ptacek et al., 1992). Interventions focused on improving coping skills, such as emotional regulation, problem-solving, and resilience, can help individuals to better manage stress and trauma-related symptoms. These skills would not only help mitigate the impact of risk factors but also foster a sense of self-efficacy and personal control, which are key aspects in preventing the development of anxiety, stress, and depression (Cicchetti & Rogosch, 2009; Sheffler et al., 2019). In this regard, previous studies had shed light on the possibility that differences in coping may exert an effect on the relative frequency with which people experience psychological disorders (Nolen-Hoeksema, 1991). Indeed, some authors have even suggested that coping tactics such as venting and behavioural disengagement can be used as “red flags” to trigger an early screening for depression in some patients (Martin et al., 2021). Therefore, expanding participants’ coping repertoires and improving the quality of their coping strategies could contribute to buffering the long-term effects of ACEs.

## Data Availability

Data available on request due to privacy/ethical restrictions.
